# Melatonin promotes apoptosis of thyroid cancer cells via regulating the signaling of microRNA-21 (miR-21) and microRNA-30e (miR-30e)

**DOI:** 10.1080/21655979.2022.2054206

**Published:** 2022-04-12

**Authors:** Hao Jia, Wei Sun, Xiangbo Li, Wenhao Xu

**Affiliations:** Thyroid & Vascular Surgery Department, Zhumadian Central Hospital, Zhumadian, Henan Province, China

**Keywords:** Melatonin, thyroid cancer, lncRNA7, microRNA, cell proliferation, cancer cell apoptosis, cell implantation

## Abstract

Melatonin (MEL) is an effective therapeutic choice for thyroid cancer treatment. In this study, we aimed to explored the potential effect of MEL upon the drug sensitivity of cancer cells and the according underlying mechanisms. Thyroid cancer mice were established as a control group and a MEL group to observe the in vivo effect of MEL. Tumor size and weight in nude mice were detected to evaluate the effect of MEL on tumor growth. Immunohistochemistry assay (IHC) and Western blot were performed to analyze the expression of PTEN protein in tumor cells or tumor cells. After 32 days of cancer cell implantation, MEL was found to significantly repress tumor growth in nude mice approximately by half. Moreover, MEL also suppressed tumor cell proliferation, while apparently activating the apoptosis of tumor cells. In addition, hydrogen sulfide (H_2_S) production was obviously elevated by MEL treatment. Mechanistically, the expression of phosphatase and tensin homolog (PTEN) was remarkably activated by MEL treatment in tumor tissues of implanted TPC-1 and BCPaP cells in nude mice. Meanwhile, MEL inhibited the expression of miR-21 and miR-30e and promoted the expression of lncRNA-cancer susceptibility candidate 7 (CASC7). Both miR-21 and miR-30e could suppress PTEN expression, while miR-21 could also inhibit the expression of lncRNA-CASC7. In conclusion, the results demonstrated that the MEL administration could downregulate the expression of miR-21 and miR-30e, which resulted in increased expression of PTEN, a pro-apoptotic tumor suppressor, to promote the apoptosis of thyroid cancer cells.

## Graphical Abstract

**Figure:**
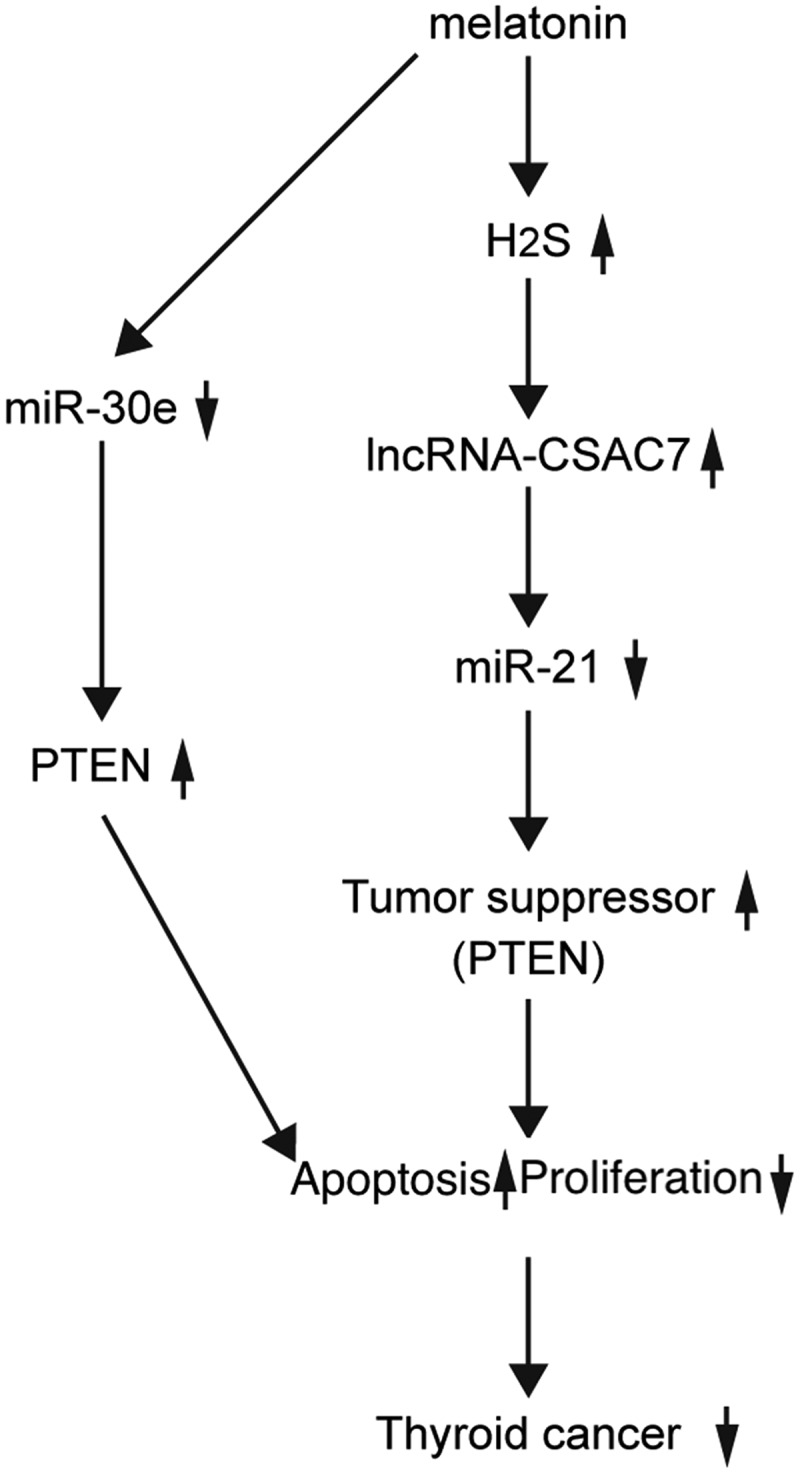

## Introduction

Thyroid cancer is one of the most frequently diagnosed endocrine cancer, with a rapidly growing yearly occurrence rate worldwide [1,2]. In 2015, about 100,000 new thyroid cancer cases were reported in China [3]. Differentiated thyroid cancer (DTC), which includes both follicular thyroid cancer and papillary thyroid cancer, accounts for most of thyroid cancer cases and typically reacts well to surgical procedure in conjunction with radioiodine (131-I) treatment [4]. On the other hand, inadequately differentiated or undifferentiated thyroid cancer such as anaplastic thyroid cancer (ATC) makes up virtually 50% of all thyroid cancer associated mortality [5].

LncRNAs are specified as a type of transcripts with no protein coding ability that contain greater than 200 nucleotides. LncRNAs were first recognized as transcriptional noises [6]. Nevertheless, current evidence shows that lncRNAs are associated with various features including epigenetic control of transcription [7]. In contrast, miRNAs are a type of tiny and single-stranded RNAs associated with the control of different intracellular activities, including cell growth, survival, proliferation, inflammatory reaction, as well as tumorigenesis [8]. LncRNAs are typically thought as noncoding RNAs (ncRNAs) which manage gene expression. For example, lncRNA-CASC7 was shown to inhibit the expansion of colon cancer cells by suppressing miR-21 expression [9]. Moreover, bioinformatic evaluation found a miR-21 binding site on CASC7 as well as on the 3-untranslated region (3’-UTR) of PTEN. As a result, in individuals with asthma resistant to corticosteroid treatment, CASC7 may target miR-21 to upregulate the expression of PTEN while raising the sensitivity level of airway smooth muscle cells (ASMCs) to glucocorticoid via hindering the signaling of PI3K/AKT.

PTEN is recognized as one of the most essential targets of miR-21 in cancer advancement, and an inverted relationship was discovered between the degrees of miR-21 expression and the intensity of PTEN signals [10,11]. The upregulation of miR-21 and the downregulation of PTEN are validated to lead to higher incidence of non-small-cell lung carcinoma (NSCLC). Therefore, these results show that miR-21 is featured as a tumorigenic element by negatively controlling the expression of PTEN in human NSCLC. The degree of miR-21 expression is also negatively associated with the degrees of PTEN protein as well as high degrees of miR-21 expression and a much shorter DFS. Certain proof has actually showed that PTEN is a gene suppressing tumor growth. Actually, homozygous PTEN deletions were seen in a number of tumors [12–14]. In addition, PTEN has the ability to reduce cell proliferation, while the increased PTEN expression prevents the transition from the S phase in cell lines derived from thyroid tumors [15,16].

It has been reported that MEL increased the production of H2S, which may upregulate the expression of lncRNA-CSAC7 to downregulate the expression of miR-21 [17,18]. Meanwhile, MEL also downregulates the expression of miR-30e [19,20]. In this study, we aimed to explore the role of MEL in the regulation of the drug sensitivity of thyroid cancer cells. As PTEN is a shared target gene of miR-21 and miR-30e, we hypothesized that MEL may suppress the proliferation and promote the apoptosis of thyroid cancer cells via regulating the signaling pathways of H_2_S/CSAC7/microRNA-21/PTEN and microRNA-30e/PTEN. To test the above hypothesis, we incubated thyroid cancer cells with MEL and then implanted those cells into thyroid cancer mice. This study for the first time purposed H_2_S as a potential factor to participate in the signaling pathway of CSAC7, and investigated two different miRNA-involved signaling pathway to study the mechanism of MEL’s therapeutic effect upon thyroid cancer.

## Materials and methods

### Cell culture and transfection

To establish a cellular model of thyroid cancer, TPC-1 (Sigma Aldrich, St Louis, MO; cat number: SCC147) and BCPaP cells (DSMZ, Braunschweig, Germany; cat number: ACC273) were kept in a DMEM medium (Gibco, Thermo Fisher Scientific, Waltham, MA) supplemented with 10% (v/v) of FBS (Sigma Aldrich, St Louis, MO) as well as Pen (5000 units/mL)/Strep (5000 µg/mL) (Gibco, Thermo Fisher Scientific, Waltham, MA). All cells were cultured at 37°C in 5% carbon dioxide and saturated humidity. When the cells reached exponential growth, they were divided into 2 group, i.e., 1. Control group (Untreated cells), and 2. 5 mM MEL group (cells treated with 5 mM of melatonin). Melatonin concentration was chosen according to previous published articles and was prepared using DMSO solution. The cells were treated under respective conditions for 48 hrs. At 48 hrs after treatment, the cells were harvested for later analyses.

### Animal model establishment

BALB/C female nude mice (about 4 to 6 weeks old) were bought from Charles River (Beijing) Laboratory (Beijing, China) and adapted to the environment in the animal facility for 7 days. Then, 1 × 10^6^ TPC-1 and BCPaP cells were diluted in 100 μl of chilled PBS (PH = 7.4) and injected subcutaneously to the right forelimb of each mouse to establish a tumor model of thyroid cancer. After the successful establishment of the thyroid cancer model, the mice were arbitrarily divided into two groups, i.e., 1. Control group (thyroid cancer mice not treated with MEL) and 2. MEL (thyroid cancer mice treated with a daily dose of 25 mg/kg of MEL for 32 days). At the same time, all mice in both groups were irradiated with a radiation dose of 2 Gy two times weekly. During the entire treatment of 25 days, the growth dimension of tumor in each mouse was detected twice a week by using a Vernier caliper, so that the tumor volume could be approximated in accordance with the formula: tumor volume = 0.5 × W × L, in which L represented the largest tumor diameter while W is the perpendicular diameter of the tumor. Throughout the radiation treatment, the mouse was placed under a plumbum mold and the tumor was exposed to the radiation ray. After 32 days of therapy in conjunction with or without MEL, the tumor tissues were taken out from dissected mice and made into paraffin embedded section for subsequent analyses. Institutional ethical committee of Zhumadian Central Hospital has approved the protocol of this study (Approval ID: ZMDCH20170731B).

### RNA isolation and real-time PCR

Collected tissue and cell samples were treated in a Trizol reagent (Invitrogen, Carlsbad, CA) to isolate the total RNA content, which was quantified and 1 μg of the total RNA from each sample was reversely transcribed into cDNA transcripts by making use of universal primers as well as a SuperScript III First-Strand Synthesis Assay Kit (Invitrogen, Carlsbad, CA) in accordance with the experimental protocols provided by the assay manufacturer. In the next step, real-time PCR was carried out on a QuantStudio 3 Real-Time PCR Instrument (Applied Biosystems, Foster City, CA) as well as a Power SYBR Green Master Real-Time PCR Assay kit (Applied Biosystems, Foster City, CA) in accordance with the experimental protocols provided by the assay manufacturer. All experimental data was evaluated by using the QuantStudio Software equipped on the QuantStudio 3 Real-Time PCR Instrument (Applied Biosystems, Foster City, CA). Finally, the expression of miR-30e (Forward primer: 5’-GTAAACATCCTTGACTGG-3’; Reverse primer: 5’-GAACATGTCTGCGTATCTC-3’), lncRNA-CASC7 (Forward primer: 5’-ATCAACGTCAAGCTGGGAGG-3’; Reverse primer: 5’-CTTGTCCCCCGCTCGTTC-3’), miR-21 (Forward primer: 5’-GCTTATCAGACTGATGTTG-3’; Reverse primer: 5’-GAACATGTCTGCGTATCTC-3’), and PTEN mRNA (Forward primer: 5’-TGAGTTCCCTCAGCCGTTACCT-3’; Reverse primer: 5’-GAGGTTTCCTCTGGTCCTGGTA-3’) in each sample was calculated by utilizing the ΔΔCt approach.

### Vector construction, mutagenesis, and luciferase assay

**Figure 7.**
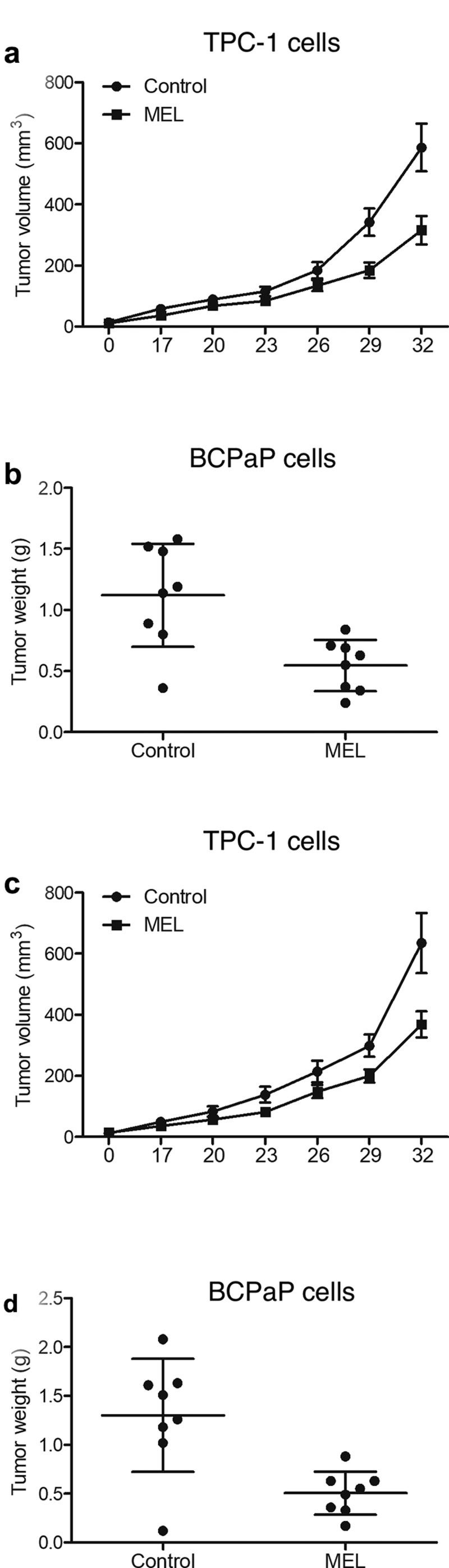
Luciferase assays showed that miR-21 and miR-30e suppressed the expression of PTEN and miR-21 while inhibiting the expression of lncRNA-CASC7 (* P value < 0.05, vs. control group).

The results of our preliminary sequence analysis showed potential binding sites of miR-21 and miR-30e in the gene sequences of lncRNA-CASC7 and PTEN, respectively. Thus, a luciferase assay was performed to explore the regulatory relationship between lncRNA-CSAC7/miR-21, miR-21/PTEN, as well as miR-30e/PTEN. At first, the sequences of CASC7 and PTEN containing respective miR-21/miR-30e binding sites were cloned and inserted into pcDNA 3.1 luciferase vectors (Promega, Madison, WI) to generate wild-type plasmids of CASC7 and PTEN, respectively. At the same time, site-directed mutagenesis (nucleotide sequence changes indicated in Figure 7a and 7d) by utilization of a QuikChange II Site-directed mutagenesis kit (Agilent Technologies, Inc., Santa Clara, CA) was carried out to mutate the miR-21/miR-30e binding sites in the sequences of CASC7 and PTEN to generate mutant-type plasmids of CASC7 and PTEN, respectively. In the next step, either mutant type or wild type of CASC7 or PTEN plasmids were co-transfected into TPC-1 and BCPaP cells (6 × 10^3^ cells per well) in conjunction with miR-21/miR-30e mimics or a scramble control by using Lipofectamine 3000 transfection reagent (Invitrogen, Carlsbad, CA) in accordance with the experimental protocols provided by the assay manufacturer. At 48 h after transfection, the cells were harvested and the luciferase activity of transfected cells was measured by using a Bright Glo luciferase assay (Promega, Madison, WI) in accordance with the experimental protocols provided by the assay manufacturer.

A: Sequence analysis indicated potential binding of miR-21 to lncRNA-CASC7.B: The luciferase activity of wild-type lncRNA-CASC7 was inhibited by miR-21 in TPC-1 cells.C: The luciferase activity of wild-type lncRNA-CASC7 was inhibited by miR-21 in BCPaP cells.D: Sequence analysis indicated potential binding of miR-21 to PTEN.E: The luciferase activity of wild type PTEN was inhibited by miR-21 in TPC-1 cells.F: The luciferase activity of wild type PTEN was inhibited by miR-21 in BCPaP cells.G: Sequence analysis indicated potential binding of miR-30e to PTEN.H: The luciferase activity of wild type PTEN was inhibited by miR-30e in TPC-1 cells.I: The luciferase activity of wild type PTEN was inhibited by miR-30e in BCPaP cells.

### Cell proliferation assay

When the cells undergoing various treatments grew to 70% confluence, they were rinsed two times by PBS, trypsinized by using 0.25% trypsin, and seeded into 96-well tissue culture plates at 6 × 10^3^ cells per well in 200 μl of cell suspension. Then, the proliferation status of the cells was measured by using an MTT assay kit (Sigma Aldrich, St Louis, MO) in accordance with the experimental protocols provided by the assay manufacturer.

### Western blot

The total protein content in collected tissue (5 mg) and cell samples was homogenized with lysis buffer using electric homogenizer and was subsequently isolated by utilizing a protease inhibitor-supplemented radioimmunoprecipitation assay (RIPA) buffer (Thermo Fisher Scientific, Waltham, MA) for 2 h at 4°C. After that, the protein content was quantified by utilizing a BCA assay kit (Thermo Fisher Scientific) in accordance with the experimental protocols provided by the assay manufacturer. Then, the protein (50 μg/lane) in each lysate sample was resolved by using 10% SDS-PAGE, blotted onto a polyvinylidene difluoride (PVDF) membrane, and blocked for 1 h at ambient temperature in Tris-buffered saline containing 5% nonfat milk and Tween-20 (50 mmol/L of Tris-HCl, 150 mmol/L of NaCl, and 0.1% of Tween-20). In the next step, the membrane was incubated at 4°C overnight with anti-PTEN antibody (dilution: 1:1000; cat no: EP2138Y; OriGene, Rockville, MD), washed with Tris-buffered saline supplemented with 0.1% Tween 20 (TBST), and then incubated for 1 h at ambient temperature with appropriate horseradish peroxidase-conjugated secondary antibodies (dilution: 1: 2000; cat no: 32,460; Thermo Fisher Scientific, Waltham, MA) before the relative protein expression of PTEN in each sample was detected by using an enhanced chemiluminescence (ECL) reagent kit (Pierce™ Fast Western blot Kit, Thermo Fisher Scientific, Waltham, MA) in accordance with the experimental protocols provided by the assay manufacturer.

### Apoptosis analysis

The proliferation of cells (6 × 10^3^ cells per well) was measured by making use of a Guava Nexin Annexin V staining assay kit (Millipore, New York City, NY) in accordance with the experimental protocols provided by the assay manufacturer on a BD FACS Caliber flow cytometer (BD, San Jose, CA).

### IHC assay

Immunofluorescence staining of PTEN protein in collected tumor tissue samples was carried out to measure the relative expression of PTEN in each sample. In brief, tissue sections (10 μm in thickness) were treated for 1 hour with PBS added with 5% BSA as well as 0.05% of Triton-X100. EDTA (Invitrogen, Carlsbad, CA) was utilized to retrieve antigen and endogenous peroxidase activity was blocked in a 0.5% hydrogen peroxide methanol solution. Then, the sections were incubated with primary anti-PTEN antibody (dilution: 1:1000; cat no: SC-7974; Santa Cruz Biotechnology, Santa Cruz, CA) for 2 h and suitable Alexa-594 labeled secondary antibodies (dilution:1:1000; cat no: SC-8432; Santa Cruz Biotechnology, Santa Cruz, CA) for 30 min consecutively. The slides were counter-stained with DAPI for 5 min and washed with PBS. Neutral resin was used as the mounting medium and the slides were observed under a Olympus fluorescence microscope (excitation: 450 nm; emission: 590 nm; Olympus Corporation, Tokyo, Japan) to evaluate the positive expression of PTEN in each sample.

### ELISA

Concentrations of H2S in various samples were assayed by using a commercially available ELISA kit (NeoBioscience, Beijing, China) in accordance with the experimental protocols provided by the assay manufacturer.

### Statistical analysis

SPSS version 20.0 was utilized for statistical analyses of all experimental data. Each experiment was repeated in triplicate and all data is presented as mean ± standard deviation. The normality test of experimental data was carried out by using an independent-sample T-test. The level of statistical significance was set to P = 0.05. All inter-group comparisons were carried out using Student’s t tests.

## Results

### MEL treatment suppressed the tumor cell growth in nude mice

In this study, we aimed to explore the role of MEL in the regulation of the drug sensitivity of thyroid cancer cells. As PTEN is a shared target gene of miR-21 and miR-30e, we hypothesized that MEL may suppress the proliferation and promote the apoptosis of thyroid cancer cells via regulating the signaling pathways of H_2_S/CSAC7/microRNA-21/PTEN and microRNA-30e/PTEN. To test the above hypothesis, we incubated thyroid cancer cells with MEL and then implanted those cells into thyroid cancer mice.

**Figure 1.**
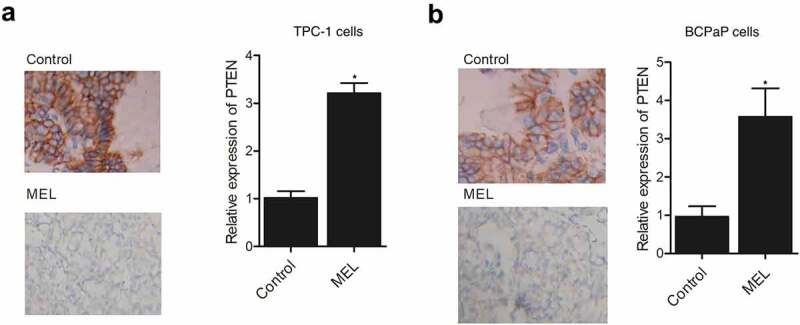
MEL treatment suppressed tumor cell growth in nude mice.

TPC-1 cells were implanted into nude mice and the growth of tumor in mice undergoing MEL treatment was monitored for 1 month. The size of tumor tissues in nude mice undergoing MEL treatment was significantly smaller than that in control mice (Figure 1a). Moreover, the weight of tumor tissues in mice administrated with MEL was remarkably decreased in comparison to that in the untreated control mice (Figure 1b). Furthermore, we implanted BCPaP cells into nude mice and observed similar results (Figure 1c, 1d).

A: The tumor size of TPC-1 cells implanted in nude mice was decreased by MEL treatment.B: The weight of tumor tissues in nude mice was decreased by MEL treatment.C: The tumor size in nude mice implanted with BCPaP cells was decreased by MEL treatment.D: The weight of tumor tissues in nude mice implanted with BCPaP cells was decreased by MEL treatment.

### MEL treatment enhanced the expression of PTEN in the tumor tissues in nude mice

**Figure 2.**
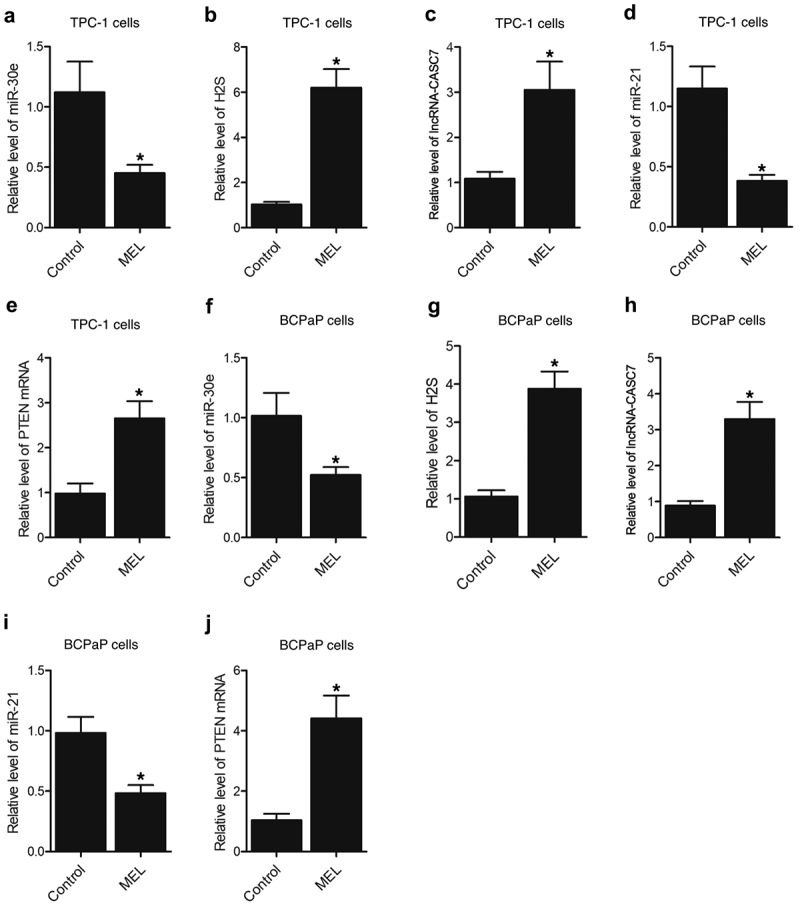
IHC analysis showed that the expression of PTEN protein in tumor tissues in nude mice was activated by MEL treatment (* P value < 0.05, vs. control group).

IHC assay was performed on the tumor tissues harvested from mice implanted with TPC-1 and BCPaP cells. The expression of PTEN was compared between the two groups. The expression of PTEN in the TPC-1 (Figure 2a) and BCPaP (Figure 2a) cells collected from MEL-treated mice was obviously elevated when compared with that in the control. These results demonstrated that the expression of PTEN was activated by MEL treatment.

A: The expression of PTEN protein in tumor tissues in nude mice implanted with TPC-1 cells was increased by MEL treatment.A: The expression of PTEN protein in tumor tissues in nude mice implanted with BCPaP cells was increased by MEL treatment.

### MEL treatment suppressed the expression of miR-30e and miR-21 while enhancing the lncRNA-CASC7 and PTEN mRNA expression along with H2S production in the tumor tissues in nude mice

**Figure 3.**
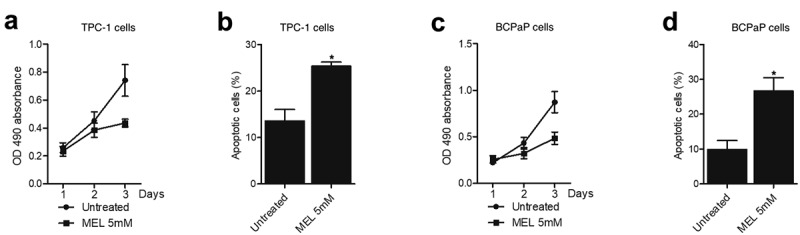
MEL treatment suppressed the expression of miR-30e and miR-21 while enhancing the lncRNA-CASC7 and PTEN mRNA expression as well as H2S production in the tumor tissues of nude mice (* P value < 0.05, vs. control group).

Furthermore, the expression of miR-30e, miR-21, lncRNA-CASC7, and PTEN mRNA as well as the H2S production was examined in the tumor tissues of nude mice. The expression of miR-30e (Figure 3a) and miR-21 (Figure 3d) was notably repressed by MEL treatment when compared with that in the control. In contrast, the expression of lncRNA-CASC7 (Figure 3b) and PTEN mRNA (Figure 3e) was remarkably elevated by MEL treatment. The production of H2S was apparently elevated in the tumor tissues from MEL-treated nude mice (Figure 3b). Meanwhile, the expression of miR-30e, miR-21, lncRNA-CASC7, and PTEN mRNA as well as the H2S production in the tumor tissues of nude mice implanted with BCPaP cells showed a similar trend (Figure 3f-j).

A: The expression of miR-30e in tumor tissues in nude mice implanted with TPC-1 cells was suppressed by MEL treatment.B: The production of H2S in tumor tissues in nude mice implanted with TPC-1 cells was enhanced by MEL treatment.C: The expression of lncRNA-CASC7 in tumor tissues in nude mice implanted with TPC-1 cells in nude mice was activated by MEL treatment.D: The expression of miR-21 in tumor tissues in nude mice implanted with TPC-1 cells was repressed by MEL treatment.E: The expression of PTEN mRNA in tumor tissues in nude mice implanted with TPC-1 cells was promoted by MEL treatment.F: The expression of miR-30e in tumor tissues in nude mice implanted with BCPaP cells was suppressed by MEL treatment.G: The production of H2S in tumor tissues in nude mice implanted with BCPaP cells was enhanced by MEL treatment.H: The expression of lncRNA-CASC7 in tumor tissues in nude mice implanted with BCPaP cells was activated by MEL treatment.I: The expression of miR-21 in tumor tissues in nude mice implanted with BCPaP cells was repressed by MEL treatment.J: The expression of PTEN mRNA in tumor tissues in nude mice implanted with BCPaP cells was promoted by MEL treatment.

### MEL inhibited the proliferation and activated the apoptosis of TPC-1 and BCPaP cells

**Figure 4.**
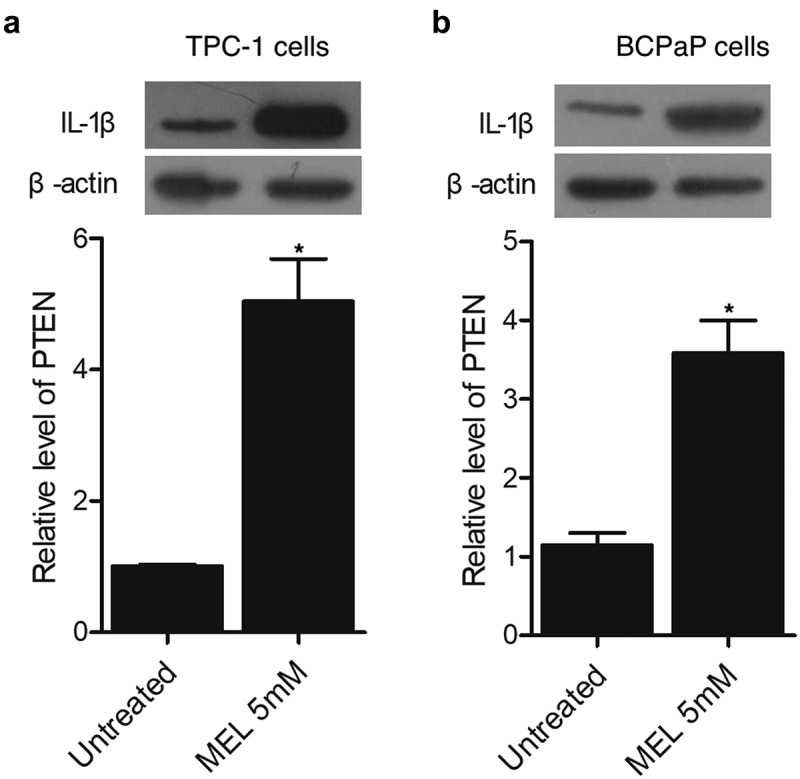
MEL inhibited the proliferation and activated the apoptosis of TPC-1 and BCPaP cells (* P value < 0.05, vs. untreated cells).

MTT assay and flow cytometry were performed to examine the proliferation and apoptosis of TPC-1 and BCPaP cells treated with 5 mM MEL. The proliferation of TPC-1 (Figure 4a) and BCPaP (Figure 4c) cells was effectively repressed by MEL treatment. Meanwhile, the apoptosis of TPC-1 (Figure 4b) and BCPaP (Figure 4d) cells was obviously activated by MEL treatment. These data suggested that MEL treatment could effectively inhibit the proliferation and promote the apoptosis of tumor cells.

A: The proliferation of TPC-1 cells was inhibited by MEL treatment.B: The apoptosis of TPC-1 cells was activated by MEL treatment.C: The proliferation of BCPaP cells was inhibited by MEL treatment.D: The apoptosis of BCPaP cells was activated by MEL treatment.

### MEL increased the expression of PTEN protein in TPC-1 and BCPaP cells

**Figure 5.**
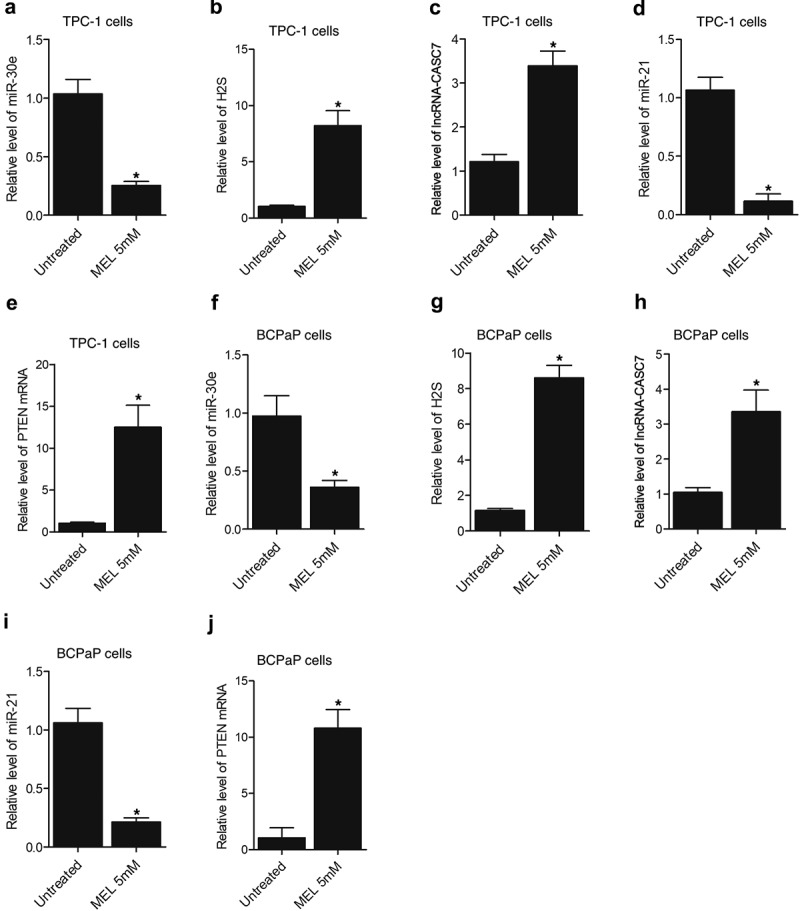
The expression of PTEN protein in TPC-1 and BCPaP cells was enhanced by MEL treatment (* P value < 0.05, vs. untreated cells).

Western blot was carried out to evaluate the expression of PTEN protein in TPC-1 and BCPaP cells treated with 5 mM MEL. The expression of PTEN protein was evidently increased in TPC-1 (Figure 5a) and BCPaP (Figure 5b) cells undergoing MEL treatment.

A: The expression of PTEN protein in TPC-1 cells was activated by MEL treatment.B: The expression of PTEN protein in BCPaP cells was activated by MEL treatment.

### MEL treatment suppressed the expression of miR-30e and miR-21 while enhancing the lncRNA-CASC7 and PTEN mRNA expression as well as H2S production in TPC-1 and BCPaP cells

**Figure 6.**
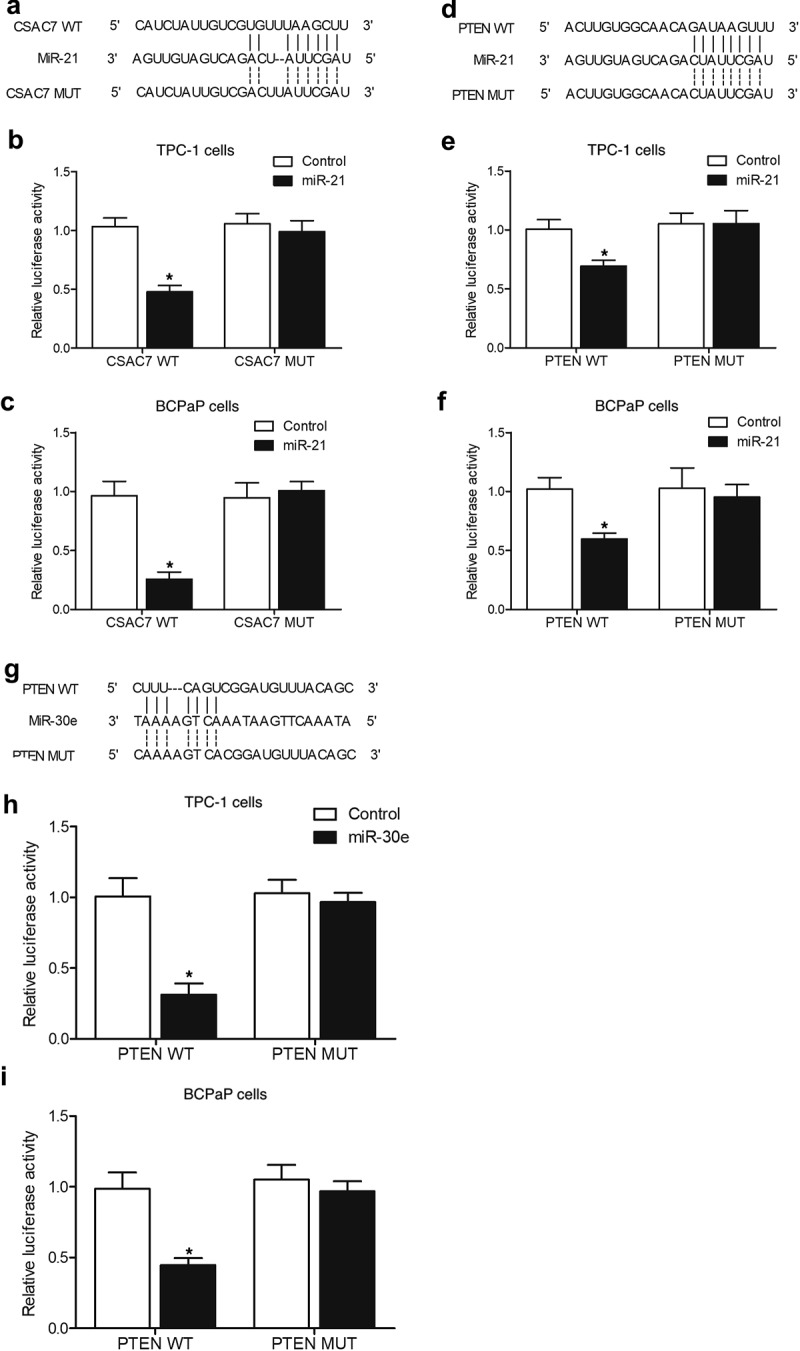
MEL treatment suppressed the expression of miR-30e and miR-21 while enhancing the lncRNA-CASC7 and PTEN mRNA expression as well as H2S production in TPC-1 and BCPaP cells (* P value < 0.05, vs. untreated cells).

Furthermore, the expression of miR-30e, miR-21, lncRNA-CASC7, and PTEN mRNA as well as H2S production was also evaluated in TPC-1 and BCPaP cells undergoing 5 mM MEL treatment. Significant inhibition of miR-30e (Figure 6a, 6f) and miR-21 (Figure 6d, 6i) expression was observed in MEL-treated TPC-1 and BCPaP cells. In contrast, the expression of lncRNA-CASC7 (Figure 6c, 6h) and PTEN mRNA (Figure 6e, 6j) was activated in TPC-1 and BCPaP cells treated with MEL. The production of H2S (Figure 6b, 6g) was also facilitated by MEL treatment in TPC-1 and BCPaP cells.

A: The expression of miR-30e in TPC-1 cells was suppressed by MEL treatment.B: The production of H2S in TPC-1 cells was enhanced by MEL treatment.C: The expression of lncRNA-CASC7 in TPC-1 cells was activated by MEL treatment.D: The expression of miR-21 in TPC-1 cells was repressed by MEL treatment.E: The expression of PTEN mRNA in TPC-1 cells was promoted by MEL treatment.F: The expression of miR-30e in TPC-1 cells was suppressed by MEL treatment.G: The production of H2S in TPC-1 cells was enhanced by MEL treatment.H: The expression of lncRNA-CASC7 in TPC-1 cells was activated by MEL treatment.I: The expression of miR-21 in TPC-1 cells was repressed by MEL treatment.J: The expression of PTEN mRNA in TPC-1 cells was promoted by MEL treatment.

### MiR-21 and miR-30e suppressed the expression of PTEN while miR-21 inhibited the expression of lncRNA-CASC7 through 3’ UTR binding

Sequence analysis showed potential binding of miR-21 to lncRNA-CASC7 (Figure 7a) and PTEN (Figure 7d), and luciferase assay was performed to explore the regulatory relationship between miR-21 and its potential targets. Luciferase vectors containing wild-type and mutant lncRNA-CASC7 and PTEN were established and co-transfected into TPC-1 and BCPaP cells with miR-21. The luciferase activities of wild-type lncRNA-CASC7 (Figure 7b, 7c) and PTEN (Figure 7e, 7f) were significantly inhibited in TPC-1 and BCPaP cells. We also found that PTEN was a potential target of miR-30e (Figure 7g). Wild type and mutant PTEN was co-transfected into TPC-1 and BCPaP cells with miR-30e. The luciferase activity of wild-type PTEN vectors was remarkably inhibited by miR-30e (Figure 7h, 7i), indicating that miR-30e suppressed the expression of PTEN.

In summary, our results indicated that MEL treatment activated H2S production and lncRNA-CASC7 expression in tumor cells, subsequently inhibiting the expression of miR-21. Meanwhile, MEL treatment inhibited the expression of miR-30e, while the down-regulation of miR-21 and miR-30e resulted in the up-regulated expression of PTEN, which in turn inhibited the growth and activated the apoptosis of tumor cells to alleviate the severity of cancer.

## Discussion

Melatonin is generated by pineal gland throughout the evening under the regulation of the body clock, the suprachiasmatic nuclei located in hypothalamus. Melatonin is likewise manufactured by immune competent cells to contribute in the monitoring as well as recuperation from infection [21–23]. The interaction in between body defense and timing is a fine tuned and controlled procedure that entails melatonin-mediated constraint of leukocyte movement from the blood flow to tissues [24,25]. Melatonin also has complex functions including the role to scavenge free radicals. It was actually been revealed that melatonin treatment can reprogram H2S pathways to avoid high blood pressure. In this study, we implanted TPC-1 and BCPaP cells in nude mice, and detected the size and weight of tumor. We found that tumor growth in MEL-treated nude mice was significantly inhibited when compared with that in the control mice. In addition, we performed qPCR, IHC and Western blot to analyze the expression of PTEN mRNA and protein in tumor tissues derived from implanted TPC-1 and BCPaP cells in nude mice that were treated with MEL. The expression of PTEN was significantly increased by MEL treatment in TPC-1 and BCPaP cells or mouse tumor tissues. It is acknowledged that BCPaP is differed from TPC-1 by the presence of a BRAFV600E mutation. However, even though we utilized these two different cells lines in our study, the presence of BRAFV600E mutation seemed to exhibit no significant influence upon our findings.

H_2_S was actually thought as the 3rd signaling molecule in the gaseous phase that exerts essential effects on the development of cancer [26–28]. Thyroid cancers are known for high rates of global occurrence [29]. A research shows that diallyl sulfide can reduce the proliferation of cells while causing their apoptosis through the mitochondrial signaling [30]. It was actually revealed that the expression of CASC7 was subdued while the expression of miR-21 as well as AKT activity was enhanced in ASMC collected from people with acute bronchial asthma. CASC7 enhanced PTEN expression by directly hindering miR-21 expression. The over-expression of CASC7 subdued the PI3K/AKT signaling while enhancing the impacts of dexamethasone on cytokine secretion through targeting miR-21.

It was actually discovered that miR-21-5p expression was low in TPC-1 cells to prevent their expansion as well as intrusion into normal tissues. Concordant with the above result, Dai et al. showed that the expression of miR-21 was dramatically reduced in people with papillary thyroid cancer [31]. On the other hand, miR-21 was upregulated in TPC-1 [32,33]. Zhang et al. confirmed that miR-21 substantially improved cell proliferation as well as metastasis via targeting programmed apoptosis 4. The deregulation of miRNAs is crucial in human PTC [33–35]. MiR‑21 transfection right into PTC led to a raised rate of cell expansion as well as a lowered rate of apoptosis [36]. MiR‑21 works by interacting with PTEN, which in turn reduces the malignancy of cancer by de-phosphorylating threonine protein kinase (Akt)/RAC‑α serine [37–39]. In another research, the downregulated miR‑21 expression influenced the Akt/PTEN signaling to inhibit the proliferation and induce G1 cycle arrest of leukemic cells in myelodysplastic syndromes [40]. In this study, we performed qPCR to evaluate the expression of miR-30e, miR-21 and lncRNA-CASC7 in tumor tissues from nude mice and in TPC-1 and BCPaP cells treated with MEL. The expression of miR-30e and miR-21 was remarkably inhibited by MEL treatment, whereas the expression of lncRNA-CASC7 was notably elevated by MEL treatment. Furthermore, we performed luciferase assay to explore the regulatory relationships between miR-21 and lncRNA-CASC7/PTEN as well as between miR-30e and PTEN. The luciferase activity of lncRNA-CASC7 was significantly inhibited by miR-21. The luciferase activity of PTEN was also significantly repressed by miR-21 and miR-30e.

PTEN works as one of miR-21 targets by de-phosphorylating its target genes [41]. PTEN mutations were actually observed in ~5% of breast cancer patients. PTEN inactivation can cause the loss of its ability as a lipid phosphatase while promoting the buildup of phosphate-dylinositol-3,4,5-trisphosphate (PIP3), leading to Akt phosphorylation [42]. It was additionally shown that the rescue of expression of PTEN in thyroid tumor subdues tumor development. Particularly, it seems that the induced expression of PTEN in thyroid tumor does not cause the apoptosis of tumor cells. Instead, PTEN obstructs tumor development via cell cycle arrest and preventing the entry of tumor cells into the S phase. Additionally, past research offered proof that p27kip1, a kinase whose function is dependent on cyclin, is involved in the role of PTEN in the progression of cell cycles. As a matter of fact, PTEN expression can up-regulate the expression of p27kip1 in thyroid cancer, while the antisense against p27kip1 eliminates the effects of PTEN on cell cycle arrest (FB-1). In this study, we performed MTT and flow cytometry assays to examine the proliferation and apoptosis of tumor cells in nude mice and in culture. The proliferation of the cells was obviously repressed by MEL treatment, while the apoptosis of the cells was apparently activated by MEL treatment.

## Conclusion

The findings of this study demonstrated that melatonin increased the production of H_2_S, which subsequently upregulated the expression of lncRNA-CSAC7 to downregulate the expression of miR-21. Meanwhile, melatonin also suppressed the expression of miR-30e, and the downregulation of both miR-21 and miR-30e resulted in increased expression of PTEN, a pro-apoptotic tumor suppressor. Therefore, we validated through the findings of this study that melatonin could promote the apoptosis of thyroid cancer cells. However, the study is limited due to the lack of clinical observations. To further validate the potential of melatonin in the treatment of thyroid cancer in patients, clinical study, as well as animal study with larger sample sized, is expected.

## Supplementary Material

Supplemental Material

## Statement

The data that support the findings of this study are available from the corresponding author upon reasonable request.
