# Supplementary figures and images for: Melatonin promotes apoptosis of thyroid cancer cells via regulating the signaling of microRNA-21 (miR-21) and microRNA-30e (miR-30e)
Source: Bioengineered. 2022 Apr 12;13(4):9588–601. doi: 10.1080/21655979.2022.2054206 (PMC9161983; doi:10.1080/21655979.2022.2054206)

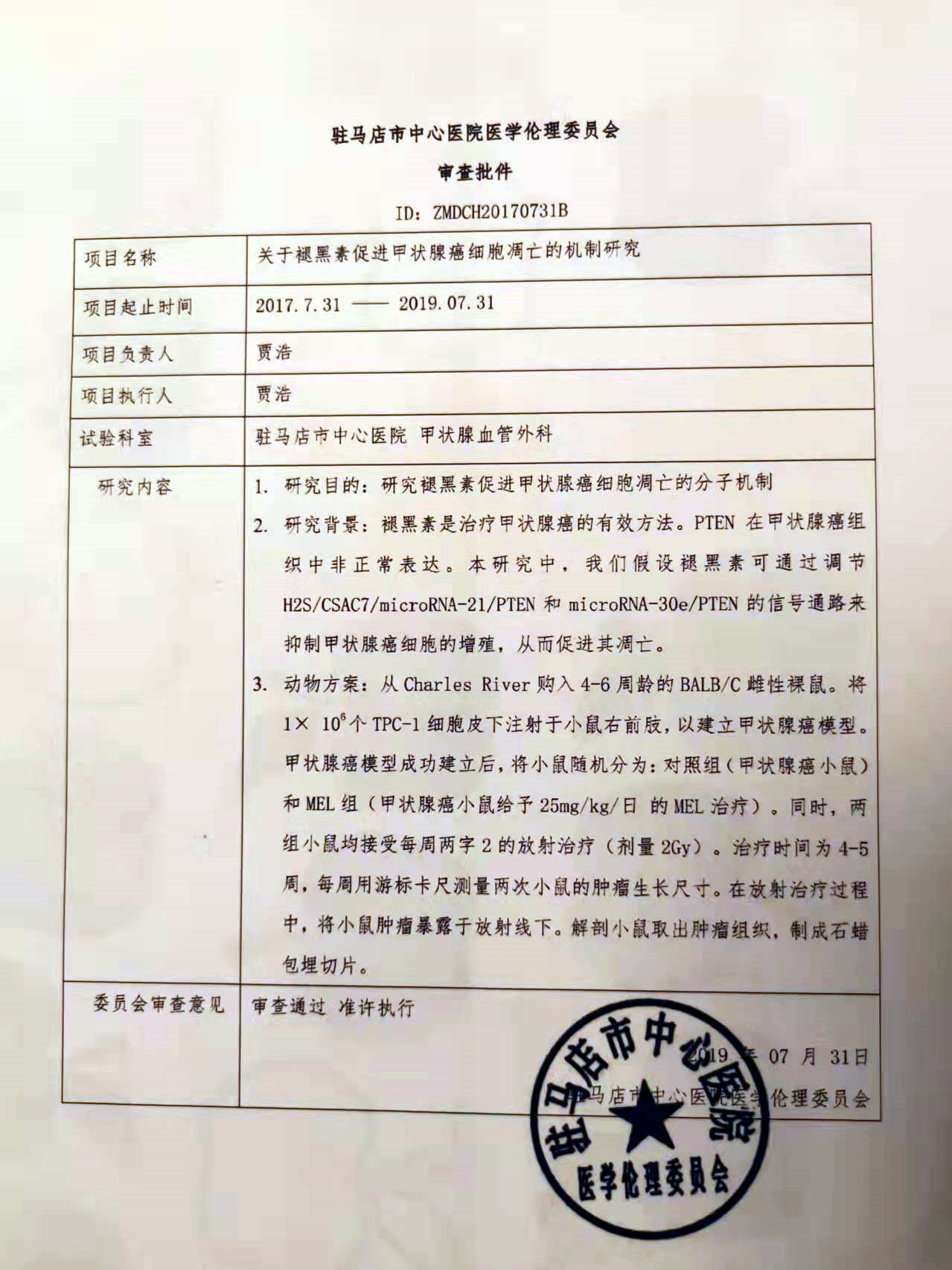

Supplement: Supplemental Material [file KBIE_A_2054206_SM2664.zip › supplementary/ethical approvement.jpg]

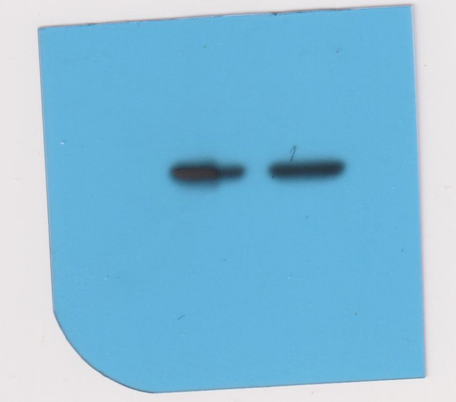

Supplement: Supplemental Material [file KBIE_A_2054206_SM2664.zip › supplementary/Fig 5A beta actin.png]

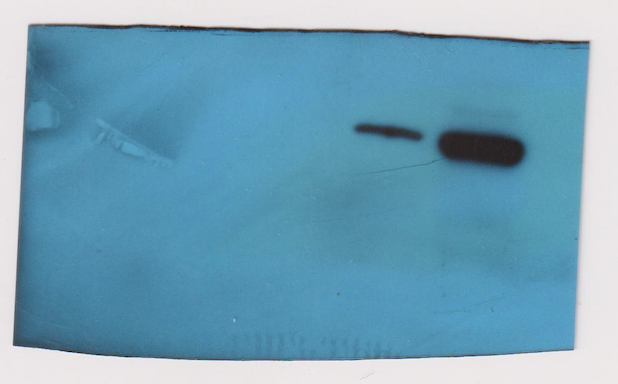

Supplement: Supplemental Material [file KBIE_A_2054206_SM2664.zip › supplementary/Fig 5A IL 1beta.png]

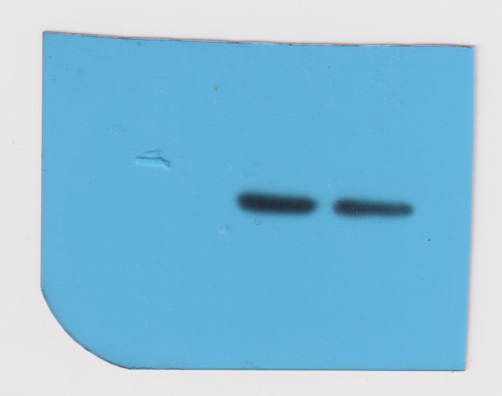

Supplement: Supplemental Material [file KBIE_A_2054206_SM2664.zip › supplementary/Fig 5B beta actin.png]

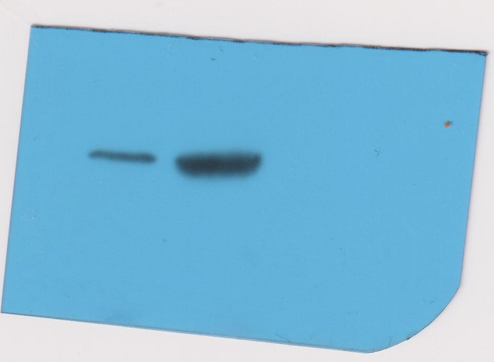

Supplement: Supplemental Material [file KBIE_A_2054206_SM2664.zip › supplementary/Fig 5B IL 1beta.png]
